# Detectable Viremia at Presentation Is a Predictor of Disease Severity in Chikungunya

**DOI:** 10.7759/cureus.58188

**Published:** 2024-04-13

**Authors:** Sumit K Rawat, Dipesh Kale, Shashwati Nema, Ram K Nema, Sudheer Gupta, Sagar Khadanga, Debasis Biswas

**Affiliations:** 1 Microbiology, Bundelkhand Medical College, Sagar, IND; 2 Microbiology, All India Institute of Medical Sciences, Bhopal, Bhopal, IND; 3 Molecular Biology, Indian Council of Medical Research (ICMR) National Institute for Research in Environmental Health, Bhopal, IND; 4 Next-Generation Sequencing (NGS) & Bioinformatics Division, 3B Blackbio Dx Ltd., Bhopal, IND; 5 Internal Medicine, All India Institute of Medical Sciences, Bhopal, Bhopal, IND

**Keywords:** poly-arthralgia, clinical severity, viral serology, viremia, molecular testing, reemerging, chikungunya virus (chikv)

## Abstract

Background

Chikungunya is a mosquito-borne re-emerging disease that has caused a significant number of outbreaks recently in diverse geographic settings across the globe. It leads to severe debilitating illness in a significant proportion of persons who are infected. Measures to limit the impact produced by recurrent outbreaks of the disease are limited and there is an urgent clinical need for early identification of those predisposed to develop severe disease. A comprehensive understanding regarding the proportion of individuals predisposed to developing severe disease is lacking as its correlation with detectable viremia is hinted at by some studies. In this context, we hypothesized that detectable viremia reflected in the diagnostic RT-PCR assay could be significantly associated with the development of severe disease in Chikungunya among those diagnosed on the basis of seroconversion. Our study aims to confirm the same in relation to disease severity among the suspected patients of Chikungunya in the setting of a tertiary care center.

Methods

In a prospective observational study at a tertiary care center, a total number of 1021 Chikungunya suspects presenting within seven days of illness were screened with Chikungunya Virus IgM ELISA from 2021 to 2023. Those having positive IgM results were further tested with RT-PCR in a blinded manner. According to the information entered into the predesigned form and the hospital follow-up/discharge data, the cases where symptoms like fever and joint pain persisted beyond two weeks were classified as severe versus those resolving within two weeks as mild. The patients in each group were compared for their clinical symptoms and association with the disease severity with detectable viremia (RT-PCR positivity).

Results

We identified a total of 178 (17.4%) lab-confirmed Chikungunya IgM-positive cases amongst the recruited patients. Here a total of 31 (18.9%) cases could be classified as severe and 133 (74.7%) as mild illness, the remaining 14 patients were excluded from analysis due to insufficient clinical data. Severe illness was significantly higher in elderly individuals belonging to more than 60 years (p = 0.01). Viremia was detected in 16 (9%), those with detectable viremia had higher odds (OR = 4.1) of manifesting as severe disease. Among the severe cases, the proportion of cases with RT-PCR positivity (8, 25.8%) at presentation was significantly higher (P = 0.01) versus those who presented with mild disease (7, 5.5%).

Conclusion

Our study reveals a correlation between detectable viremia in Chikungunya virus (CHIKV) patients and an increased risk of manifesting into a severe disease, where severe cases exhibited a significantly higher proportion of viremia, indicated by RT-PCR positivity. This study hints at the presence of viremia, joint symptoms, and elderly age as potentially useful clinical predictors of disease outcomes, these may serve as indicators for closer monitoring among individuals seeking medical attention due to Chikungunya infection. However, we need to validate these findings in future longitudinal studies incorporating multiple, time-bound follow-up data on clinical outcomes, viral titers, and its long-term complications.

## Introduction

Chikungunya which is caused by Chikungunya virus (CHIKV), has been listed as a re-emerging viral disease [1], that has caused a significant number of outbreaks recently in diverse geographic settings across the globe. Initially limited to a few countries in Africa and Asia, the disease has now been detected in over 110 countries with over 2 million cases being reported since 2005 [1]. In India alone, in the last three years, there has been an annual incidence of around 100,000 suspected and approximately 10,000 confirmed cases [2].

The clinical presentation of this condition covers a wide spectrum of severity, ranging from asymptomatic infections resolving usually within two weeks through debilitating illnesses with prolonged backache and severe arthralgia involving joints of hands and feet [3]. While the clinical determinants of the disease severity have not been clearly elucidated, early identification of individuals, vulnerable to develop severe manifestations, can lead to their prioritization for closer monitoring and enable disease mitigation strategies like rheumatic consultation, physiotherapy, etc. to limit adverse prognostic outcomes [4]. Some studies have suggested high levels of inflammatory markers and inadequate immune responses co-relate with severe disease [5,6]. While, some other studies have hinted at increased viral replication resulting from the inadequate protective immune responses as a pathogenic mechanism responsible for the development of severe disease characterized by prolonged articular manifestations [7], which appears to be mechanistically mediated by the exaggerated inflammatory immune response triggered by the unrestricted viral replication [7,8].

Given the above, and considering the clinical need for identifying reliable predictors for severe disease in Chikungunya, we hypothesized that detectable viremia reflected in the diagnostic RT-PCR assay could be significantly associated with the development of severe disease in Chikungunya. Thus among this subset, we conducted the present cross-sectional study with the objective of ascertaining if individuals with detectable viremia were more likely to experience severe manifestations of Chikungunya as compared to the ones without detectable viremia.

## Materials and methods

This prospective observational study was performed in the setting of a tertiary care teaching hospital located in Central India from 2021 to 2023, during which we recruited consecutive patients attending the medical outpatient department with fever for less than seven days’ duration. Cases already on treatment or those in whom alternative diagnosis had already been established were excluded.

The study protocol was approved by the institutional review board and the institutional human ethics committee (reference no. 2019/PhD/Jan/19/11), and the patients were recruited into the study after obtaining written informed consent. The clinical details of the recruited participants were recorded in a pre-designed case record form (See Appendix). Patients in whom fever and arthralgia persisted beyond two weeks were classified as suffering from severe disease as evidenced by the case records and the hospital admission records [3].

Whole blood samples were collected from the recruited patients and transported to the Regional Virology Lab within the Department of Microbiology of the same hospital maintaining a cold chain as per the operational procedures for the viral diagnostic lab under the aegis of the Department of Health Research (DHR), Government of India [9].

The serum was separated and tested for CHIKV IgM antibodies using the kit manufactured by the National Institute of Virology (NIV, Pune, India), according to the manufacturer’s instructions [10]. IgM antibodies in the patient's serum (if present) and IgM from Positive Control (PC) are captured by anti-human IgM (µ chain specific) coated onto the solid surface (wells). In the next step, CHIK antigen (inactivated chikungunya virus) is added which binds to captured human chikungunya-specific IgM. The washing step takes care of the unbound antigen. In the subsequent step, biotinylated anti-CHIK monoclonal antibodies are added. Avidin-HRP was added after washing and finally, the chromogenic substrate (TMB/H2O2) was added, followed by stop solution (1N H2SO4). The optical density (OD) was measured at 450 nm and for interpreting the results following were used: IgM Negative: If sample OD ≤2×OD of Negative Control (NC). IgM Positive: If sample OD ≥3×OD of NC. Equivocal: If sample OD ≥2×OD of NC but ≤3×OD of NC.

Viral RNA isolation and RT-PCR

RNA extraction was done using 140 µl of serum with the spin-column method using the QIAamp Viral RNA Mini Kit (Qiagen, Hilden, Germany) as per the instructions supplied by the manufacturer [11]. We initially incubated the mixture at room temperature for 10 minutes to ensure complete virus lysis and performed centrifugation in a cooled centrifuge at 6000 x g.

The Real-time PCR (RT-PCR) test was performed on the extracted RNA by TRUPCR® Chikungunya Real-Time PCR kit (3B Blackbio Dx Ltd., India) from samples that tested positive for CHIKV IgM. The kit targets highly conserved regions of the E1 region in CHIKV genome in an in-vitro nucleic acid amplification assay and is a one-step RT-PCR assay in which RNA templates are first reverse transcribed to generate complementary cDNA strands followed by DNA polymerase-mediated cDNA amplification. The reaction was performed with a final reaction mix volume of 15 µl and 10 µl of RNA. The assay involved the target fluorophore labels using FAM for the Chikungunya E1 gene (expected Ct: ± 20) and Texas Red for Internal control (Expected Ct: ± 20). The total number of cycles was set to 45 whereas the cut-off for any positive sample was set to 40.

Statistical analysis

Data analysis was conducted with the GraphPad QuickCalcs free version. The test of statistical significance for differences between two independent proportions was assessed using Fischer’s Exact test and the same for two means was ascertained by unpaired t-test, p <0.05 was considered statistically significant. The odds ratios (OR) were calculated as per conventional definition with a confidence interval (CI) of 95%.

## Results

**Figure 1:**
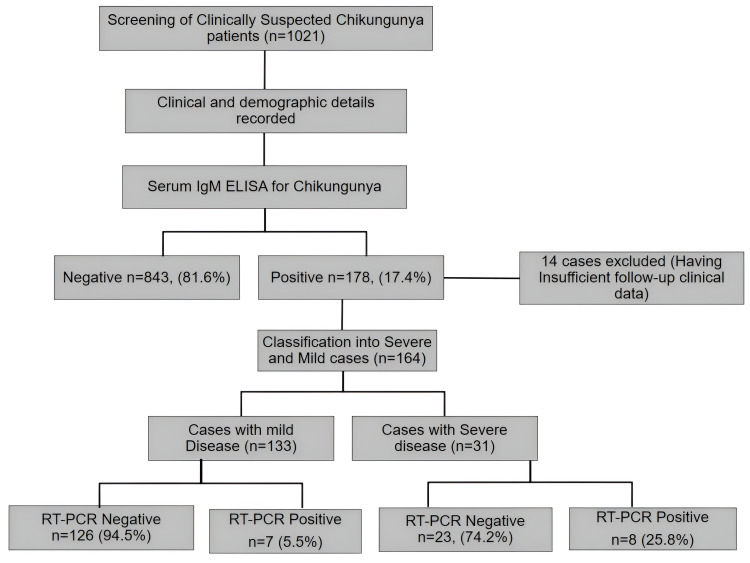
Workflow of the study

A total of 1021 patients (586 males) were recruited into the study. The study participants' mean (± SD) age was 29 (± 13) years. As shown in Figure 1, all the Chikungunya suspects were tested for the presence of IgM antibodies to CHIKV, and 178 (17.4%) tested positive for the same. We had already excluded four patients, where two of these had co-infection of dengue and two were undergoing treatment for tuberculosis.

The predominant manifestations among the recruited patients were fever (100%), malaise/weakness (68.3%), arthralgia (57.8%), myalgia (51.2%), chills (32.1%), headache (20.7%), sore throat (11.5%), retro-orbital pain (9.5%), joint swelling (7.9%) and diarrhea (3.4%). The mean (± SD) duration of fever among the recruited patients was 3 (±2) days. The comparative profile of the demographic features and clinical manifestations of the Chikungunya suspects and Chikungunya-positive participants is shown in Table 1. The mean (± SD) duration of illness in the seropositive patients was 6 (± 5) days.

**Table 1:** Comparison of clinical manifestations among the Chikungunya suspects and the serology-positive patients * p is significant (s)

	Chikungunya suspects (n = 1021)	Chikungunya positive (n = 178)	p value
Age (Mean ± SD)	29 (± 13)	31 (± 13.8)	0.062
Gender (M : F)	1.35 : 1	1.86 : 1	0.026*
Fever (> 100°F)	972 (95.2%)	171 (96.1%)	0.953
Chills	589 (57.7%)	79 (44.4%)	0.079
Rigor	521 (51.0%)	61 (34.3%)	0.001*
Malaise / weakness	498 (48.8%)	70 (39.3%)	0.163
Joint pain	487 (47.7%)	118 (65.7%)	0.012*
Myalgia	455 (45.6%)	92 (51.7%)	0.317
Rashes	236 (23.1%)	32 (18%)	0.247
Headache	212 (10.5%)	26 (14.6%)	0.127
Vomiting	114 (11.2%)	23 (16.3%)	0.529
Retro-orbital pain	69 (6.7%)	11 (6.1%)	0.872
Joint swelling	59 (5.8%)	19 (10.7%)	0.037*
Cough	58 (5.7%)	7 (3.9%)	0.471
Diarrhea	49 (4.8%)	9 (5.1%)	0.850
Conjunctivitis	37 (3.6%)	2 (1.1%)	0.106

**Figure 2:**
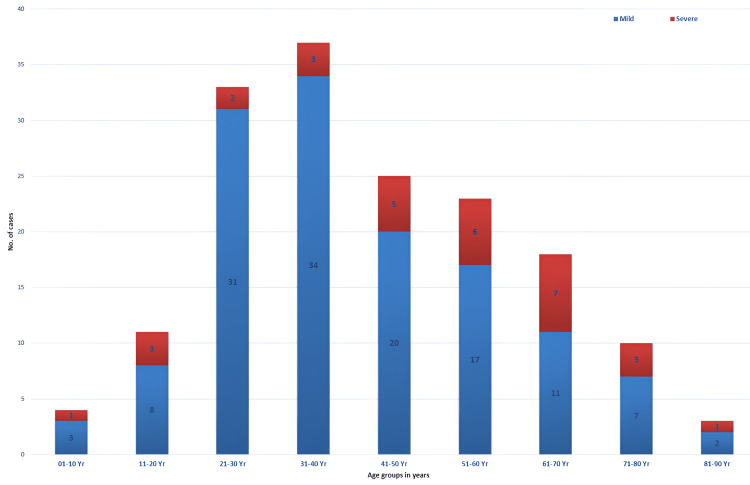
Age group-wise distribution of mild and severe Chikungunya cases (Total, n=164)

We next categorized the seropositive patients into mild (n=133) and severe cases (n=31), based on the persistence of articular manifestations beyond two weeks. We excluded 14 patients from the analysis due to insufficient clinical data. While the majority of the seropositive patients belonged to the age group of 31-40 years, severe illness was significantly higher in the individuals aged more than 60 years (p= 0.01) (Figure 2).

While most of the clinical features were similarly distributed across the severity spectrum, joint pain (p=0.02) and joint swelling (p=0.03) were observed more commonly in severe cases as seen in Table 2.

**Table 2:** Frequency of presenting symptoms in the two groups (mild and severe cases) * P is significant (s)

Clinical Symptoms	Mild cases, Total n=133 (%)	Severe, Total n=31 (%)	P Value
Fever	130 (97.7)	31 (100)	1
Myalgia	89 (66.9)	25 (80.6)	0.2
Joint Pain	90 (67.7)	28 (90.3)	0.02*
Rashes	26 (19.5)	6 (19.3)	0.9
Headache	19 (14.3)	2 (6.5)	0.4
Joint swelling	11 (8.3)	8 (25.8)	0.03*
Diarrhea	6 (4.5)	3 (9.8)	0.4

Furthermore, exploring the association of detectable viremia with disease severity, we observed a higher proportion of RT-PCR-positive viremic individuals among the severe cases (25.8% vs 5.5%) (p=0.01; OR=4.01 (95% CI=1.36-11.83)).

## Discussion

In this study, we report the clinical profile of Chikungunya patients among the cohort of febrile patients reporting to a tertiary care teaching hospital located in the central part of India over three years. While we observed significant overlapping of clinical manifestations between febrile patients with and without Chikungunya, features like joint pain, swelling, and elderly age were significantly over-represented in the severe cases of this disease. Furthermore, the proportion of viremic individuals, as reflected by RT-PCR positivity, was significantly higher in the severe cases.

Considering the endemicity of Chikungunya in tropical countries [12], its wide spectrum of clinical severity, and overlapping clinical presentations with other febrile illnesses, the identification of features associated specifically with the severity of the disease is a clinical necessity [13]. Our findings identifying the predominance of joint pain, joint swelling, elderly age, and RT-PCR positivity as the correlates of severe disease, could have implications in clinical decision-making by assisting in prioritizing patients requiring closer monitoring. In addition, the association of RT-PCR positive status in a significantly higher proportion of severe cases also hints at a potential mechanistic basis for the pathogenesis of disease severity. As reflected by RT-PCR positivity, increased viremia in these individuals could trigger increased systemic inflammation, driving the articular manifestations observed clinically in severe cases. Though these correlates need to be validated in larger multi-centric studies, to our knowledge, this is the first such report suggesting potential identifiers of clinical severity in Chikungunya.

Similar to our findings, previous studies conducted in other Asian countries like India, Pakistan, Thailand, and Indonesia have also reported a preponderance of male gender in Chikungunya [4,14-17]. However, a study conducted on the 2005 outbreak of Chikungunya in the Reunion Islands reported a higher incidence of female patients [18]. While this could be an isolated finding in that particular outbreak, most of the studies in endemic countries across the world are in agreement with the male predominance observed by us. Likewise, severe illness was reported in patients above 60 years of age in several earlier studies conducted in various countries from Asia and Africa [4,7,8,14-18]. Our findings on the increased prevalence of arthralgia and arthritis in severe cases are also in sync with previous studies reported in the literature [6,14,18,19-21].

Our study suffered from two major limitations. Being a hospital-based study, our patients are not exactly representative of the general population of Chikungunya patients, who predominantly seek care at primary and secondary-level healthcare facilities. This selection bias might have skewed the observed proportion of severe cases. Secondly, being a cross-sectional study, we could not follow up on the long-term outcomes of the patients categorized as severe cases, the duration of viremia too could not be ascertained and compared between the mild and severe cases through sequential sampling.

## Conclusions

Though this study hints at the presence of viremia, joint symptoms, and elderly age as potentially useful clinical predictors of disease outcomes in Chikungunya, we need to validate these findings in future longitudinal studies incorporating multiple, time-bound follow-up data on clinical outcomes, viral titers and long-term complications of this condition. It would also be clinically insightful to elucidate the immunological basis of disease severity through a comparative assessment of key immune mediators between patients with mild and severe disease.

## Figures and Tables

**Figure 3:**
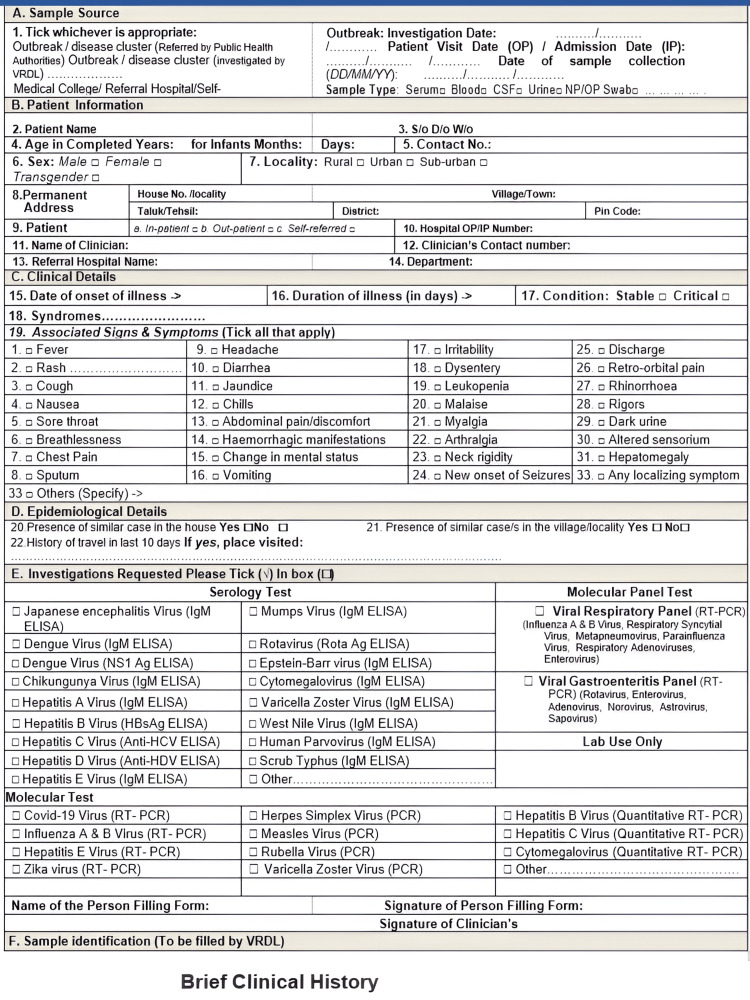
Test requisition and case record form

## Appendices

Appendix 1: Figure 3 shows the requisition/case record form used for the study.
